# “Good food equals good health”: a focus group study of adolescent boys’ perceptions of eating and weight

**DOI:** 10.1186/s12889-024-17740-6

**Published:** 2024-01-22

**Authors:** Erika Hansson, Manuela Schmidt

**Affiliations:** 1 Faculty of Education, Kristianstad University, Kristianstad, Sweden; 2 Department of Quality Improvement and Leadership, School of Health and Welfare, Jönköping University, Jönköping, Sweden

**Keywords:** Adolescent boys, Disordered eating, Eating, Focus group, Weight

## Abstract

**Background:**

Disordered eating refers to unhealthy, sometimes excessive eating including so-called compensatory behaviours such as extreme dieting or voluntary vomiting. Between 24% and 30% of adolescent boys are suggested to engage in disordered eating, making it a significant public health issue. However, current instruments for assessing disordered eating among adolescents have been primarily developed and validated for girls and women, which may make for flawed assessment of boys. The aim of this study is to shed light on adolescent boys’ perceptions of eating, weight, and food intake to better understand their perspectives in service of disordered eating research.

**Methods:**

This exploratory study was conducted from May to November 2022 using focus groups with a total of 39 adolescent boys (aged 12–19 years) who attended 7th to 12th grade in one of four schools in Southern Sweden. In addition, participants completed a form with questions on background demographics and eating habits. The transcripts of the focus group discussions were analysed using thematic analysis.

**Results:**

The quantitative data showed that around one third of the adolescent boys were overweight or obese. They ate at least one meal per day with the family and ate healthy food about five days per week and unhealthy food about three. Analysis of the qualitative data yielded six themes: *The intertwined relationship between food and one’s health*, “*Don’t worry, food makes you happy,”* “*To be hungry or not. That is the question,” Boys DO care about appearances*, *Dieting and weight gain*, and *Disordered eating is a tricky matter*.

**Conclusion:**

Adolescent boys appeared to have a good understanding of food and healthy eating. They also experienced body positivity and seemed to have only minor issues regarding their weight. The primarily pathological perspective used to measure disordered eating among girls seems in need of revision to adhere to boys’ thoughts and ideas regarding eating and weight.

*Disordered eating* refers to unhealthy, sometimes excessive eating including so-called compensatory behaviours such as extreme dieting or voluntary vomiting [1–3]. However, such compensatory behaviours are not frequent or severe enough to render an eating disorder [ED] diagnosis according to the Diagnostic and Statistical Manual of Mental Disorders (5th ed.; DSM–5; American Psychiatric Association, 2013). Although there is a large over-representation of girls among those presenting with disordered eating (and ED) [4], around 24% of the adolescent male population [5]– upwards of 30% in a recent study by Nagata, Garber [6] or even as many as half of the population of adolescent boys [7]– are estimated to engage in disordered eating. As such, disordered eating a public health issue of particular note [8, 9], not only because it concerns the urgency of reducing adolescent suffering but also consideration of how such experiences in adolescence could impact the individual later in life. Disordered eating is a predictor of an ED [10, 11] and could prove fatal if untreated [12]. While volatility in the definition of disordered eating can obscure potential relationships with other constructs, studies point to correlations between disordered eating and low self-esteem [13, 14], depression [15], emotional dysregulation [16, 17], negative body perception [18], social anxiety [19], and suicidal ideation [20, 21]. Again, the potential individual and societal impacts on the general health of youth in general and adolescent boys in particular are vast.

Despite the estimated high numbers of adolescent boys with disordered eating, current instruments that measure and assess EDs and disordered eating among adolescents have primarily been developed and validated for girls and women between the ages of 18 and 25 years [22, 23]. Such instruments may not work optimally for boys [24] as gender appears to play a role in how disordered eating is expressed [25]. Unique characteristics for boys are suggested to be weight gain behaviours driven by an ideal body characterized by muscularity and leanness [26, 27], which conflicts with questions in the instruments that describe perceptions specific to girls such as “I think that my thighs are too large” or “hips are too big” [28]. For this reason, gender should be considered an important factor when examining the maintenance and aetiology of disordered eating [25, 29], especially in non-clinical settings [30]. Due to the lack of valid instruments for measuring boys’ disordered eating, as the diagnostic criteria assume that the same symptoms apply to both genders [32], research on adolescent boys is scarce [7], with figures being underestimated [31] and potentially skewed [25]. A parallel could be drawn to the research on attention deficit hyperactivity disorder (ADHD), where girls show somewhat different behaviour than boys despite having the same basic symptoms, which demands other methods to confirm a diagnosis [32].

Clearly, the trajectories of adolescent boys’ development of disordered eating and what disordered eating is to them is worth exploration but would require instruments that are valid for this group. Therefore, the aim of this study is to shed light on adolescent boys’ perceptions of eating, weight, and food intake to contribute to further understanding of this understudied perspective of boys.

## Method

The present study employed an exploratory research approach utilizing a focus group data collection method [33]. Given its exploratory nature, the study centred on understanding the human experiences, perceptions, and feelings related to food intake, disordered eating, and body weight among adolescent boys.

### Participants

For practical reasons, we chose a convenience sample of four different schools in Southern Sweden, where teachers were contacted, and the research was permitted by the principals. Three public schools and one charter school was included. The inclusion criteria for participation were (1) male gender, (2) being between 12 and 19 years old, and (3) enrolled in the Swedish schooling system. No exclusion criteria were applied. Most students had Swedish as their first language, while several had a different mother tongue. However, the boys possessed adequate language skills in Swedish, enabling them to comprehend the voluntary participation requirements and actively engage in the focus group discussions. Written informed consent was obtained from all participants and from caregivers when participants were under the age of 16. In total, 39 boys (M_age_= 15.5 years SD = 2.02; Median_age_= 16) agreed to participate and were included in the study.

### Data collection

The data was collected during May to November 2022 by face-to-face focus groups consisting of 3–8 boys. In a unique circumstance, an individual boy was interviewed separately as the other prospective participants were unwell. Rather than cancelling the interview, it was carried out for ethical considerations and incorporated into the study. The focus groups were conducted by the first author physically at the schools and the second participating via Zoom. A semi-structured guide, also called questioning route, was used to collect data. This guide allowed for flexibility during the conversation and to keep the flow while also fostering consistency across focus groups [33]. The guide was tested on two pilot focus groups who were not enrolled in the present study.

The focus groups took place at the participants’ schools during the school day. First, the boys were welcomed into the classroom, where the researchers presented themselves and their work. Participants then completed a form in the classroom including the participants’ self-reported height, weight, age (year and month born), and questions on level of physical activity/inactivity, and meal patterns (e.g., number of breakfasts per week 0–7). Talking was allowed and encouraged to some extent during form completion, with many boys commenting on how often they sat still (which they did mostly due to gaming). After the form was completed and collected by the first author, the focus group proceeded with the interview guide, covering various topics: (1) thoughts on health; (2) thoughts, feelings, and experiences regarding what, how, and when to eat; (3) thoughts on healthy/unhealthy eating/food; (4) thoughts and experiences of hunger and food saturation; and (5) thoughts and experiences on appearance and physical activity. The focus group interviews lasted on average 31 min (ranging from 21 to 54 min). All focus groups were transcribed verbatim.

### Analysis

The data from the form underwent analysis using descriptive statistics, computed with Jamovi [34, 35]. The data from the focus group interviews was analyzed using thematic analysis as described by Braun and Clarke [36, 37], following the six phases of (1) familiarization, (2) code generation, (3) theme construction, (4) theme review, (5) theme definition and naming, and (6) report production. The analysis started out by the authors reading and re-reading the material, including their own notes and any impressions written down during and after the focus groups. Subsequently, both authors collected data extracts and coded the material inductively and independently. The codes were then grouped together according to patterns of meaning in the data to generate preliminary themes. This phase started out independently, but the authors then compared preliminary themes they had generated. The authors worked together to review the themes by exploring the hierarchy of the codes and preliminary themes as well as refining the internal homogeneity and external heterogeneity of the data, which smoothly transitioned to the fifth phase: identifying the essence of and narrative within each theme. The final names for the themes were the consensus of both authors. In the final phase, the first author produced a first draft of the results, which was then discussed by both authors. Quotes were chosen to represent each theme.

We applied a contextualist method based on the assumption that the adolescent boys’ perceptions and experiences were integrated and influenced by their surroundings, such as the family, school setting, and their closer social interactions, as well as the wider social context, such as their use of social media. During analysis, we considered this context and its embeddedness in the adolescent boys’ meaning making of their experiences of eating. To this end, in the final discussion on the themes, we applied Banduras’ Social Cognitive Learning Theory as a framework for the discussion as the theory highlights the interactions between people, behavior and environment [38, 39], reasoning that the adolescent boys, like most people, might not always behave as they say and also to bring forth the school environment as an important place in regards to eating and nutrition.

Qualitative research, according to Braun and Clarke, is a creative and reflexive process that views the researchers’ subjectivity as a resource [40]. At all times, we sought to critically reflect on the research process, chosen methods, and our role as researchers. Awareness and reflection were included in our pre-understanding: The first author has a background in the research field of disordered eating and has worked as a teacher, while the second author has a background in health science and has conducted several focus group studies. Both authors were also the parents of boys attending school in pre-teenage age (at different schools than included in the data collection). Both authors are familiar with both qualitative and quantitative research.

### Ethics

The study was carried out in accordance with relevant guidelines and regulations according to the Declaration of Helsinki and Swedish law regarding research involving human subjects [41]. All participants (and for those under the age of 16, their caregivers) were given detailed information about the study and the e-mail addresses of researchers in case they needed to ask any additional questions. They all gave their written informed consent prior to the focus group interviews. At the start of each interview, the authors stressed the voluntary nature of participation and the right to withdraw at any given moment without providing a reason. The authors also informed participants that the data would be stored safely, and that the data would be treated confidentially. The data was analysed at the group level. The authors paid close attention to the emotional well-being of the boys during the interviews and were ready to provide necessary support. Contact details for the authors were shared in both the participants’ and caregivers’ information letters. Additionally, the participating teachers were instructed to remain vigilant and report any signs of distress or expressed concerns following the focus group sessions. Prior communication with the school nurse was also conducted.

All methods were carried out in accordance with relevant guidelines and regulations according to the Declaration of Helsinki. Ethical approval for the study was given by the Swedish Ethics Review Committee (Dnr 2,017,994). Although Swedish law dictates caregiver consent until the age of 15, we raised this age limit to 16 years for this study to create equal conditions within the classes (as 9th grade is attended by both 15- and 16-year-olds).

## Results

### Results from the form

Almost one third (*n =* 10) of the participants who answered questions regarding height, weight, year, and month of birth (*n* = 35) (four missing) were overweight and five obese according to the iso-BMI scale, which adjusts a child’s body mass index for gender and age [42]. The iso-BMI is not a gold standard but still gives an idea of the diversity of the participants.

The participants were involved in high-intensity physical activities, described as ‘difficult to communicate due to heavy breathing,’ for approximately seven hours per week on average (M = 7.11). However, there were significant variations within the group (SD = 4.87), ranging from zero hours as the minimum for high-intensity exercise to one boy claiming eighteen hours per week. The frequency of engaging in sedentary activities like reading a book or playing video games ranged from a minimum of one hour to a maximum of twelve hours daily (M = 6.09, SD = 3.40).

The participants reported eating healthy foods almost five days per week (M = 4.97, SD = 1.73) and unhealthy foods merely three days per week (M = 3.14, SD = 1.84). Almost all boys ate one meal (or at least part of it) per day with their family (M = 6.24, SD = 1.25). Table 1 shows the frequency of meals in days per week.

**Table 1:** Meal frequency in days per week (0–7) (School Lunch 0–5) (*n* = 39)

Meal	Breakfast	Lunch	School Lunch	Dinner
Mean (SD)	5.15 (2.43)	6.38 (1.65)	4.33 (1.71)	6.64 (1.14)
Median	7	7	5	7
Min/Max value	0/7	0/7	0/5	2/7

### Results from the focus groups

The thematic analysis of the adolescent boys’ perceptions on eating, body weight and food intake yielded six themes: (1) *The intertwined relationship between food and one’s health;* (2) *“Don’t worry, food makes you happy”* (3) *“To be hungry or not. That is the question”. (4) Boys DO care about appearances; (5) Dieting and weight gain; (6) Disordered eating is a tricky matter*. Quotations from the focus groups are marked “Fg X”.

### The intertwined relationship between food and one’s health

Several boys explicitly linked food with overall health, expressing the idea that consuming nutritious food was essential for maintaining good health. They perceived that *“Good food equals good health”* (Fg A). Participants noted that food was good for both physical and mental health *– “being energized in the head”* (Fg E)– and they perceived that to remain healthy one needs to eat healthy. Some also pointed to the importance of drinking water to keep healthy and having enough energy to engage in leisure activities.

What was considered good health was repeatedly debated, with much of the focus being given to physical health. For the participants, this meant not being (physically) ill, having no symptoms of coughing, avoiding COVID-19, or “*having your regular body temperature”* (Fg B). Not having any injuries and being healthy meant being able to do what you wanted without any obstacles or restrictions and be physically active: *“I can go for a run”* (Fg D). Health was discussed not only in the short-term either– participants were aware of its importance for one’s future: *“…You get a better life, yes. Yes, so you live longer, they say, if you have a healthy and strong life.”* (Fg F).

Conversely, mental health was not discussed until participants were explicitly asked about it. Participants’ knowledge of mental ill health also differed vastly across focus groups. One group (consisting of younger adolescents) discussed whether particular groups– such as bullies, people who spread rumours, people with Downs syndrome, or those who burn the Qur’an (based on current events)– were considered to have a mental illness and whether mental illness referred to an ‘illness of thoughts’. In other groups, psychological health was interpreted as not being stressed and feeling calm. Several participants spoke of the importance of not being ‘off’ or depressed, with physical and mental health being equally important. They also pondered that mental illness might not show in some people and spoke of sadness. The boys felt that friends and a well-functioning social environment were prerequisites for mental health. Reference to food was often made when talking about physical and mental health:

*“… Ill, yes, if you just look at your phone and kind of eat food. In the room, yes*

*Interviewer: Ill in what way?*

*Mentally*

*Interviewer: Yes*

*And physically.”* (Fg H)

### “Don’t worry, food makes you happy!”

*“Happiness”* (e.g., Fg D) was by far the most common association when the boys were asked about their feelings and thoughts concerning food. Others were love, joy, and life.

In general, the boys’ perceptions and experiences towards food and eating were very positive. Some longed for lunch all day and most ate what was served unless the food tasted bad. They strongly believed that eating food they did not like the taste of, or “*bad*” food, resulted in bad feelings. For example, several boys stated that mushrooms gave them bad feelings. However, in general, they expressed that they ate everything, and they also seemed knowledgeable regarding certain vitamins and proteins. Some talked about sugar and sweets, knowing that these foods were not healthy for them and of trying to eat as little as possible. One participant made a rather dramatic (albeit true) statement: *“If I don’t eat, I die!”* (Fg D).

However, happiness was easier to achieve when eating unhealthy foods: “*I like sweets, but I know they are bad for me.”* (Fg A). McDonald’s was mentioned as a physically unhealthy food, but participants reported that they seldom ate there. In general, all participants stated that they seldom indulged in unhealthy eating habits. However, they also expressed uncertainty about what constituted ‘unhealthy’ foods, engaging in discussions that compared different foods relative to their perceived healthiness. For instance, they deliberated whether certain items like meatballs and pasta could be deemed as acceptable choices, considering factors such as the degree of food processing; for example, whether the meatballs were prepared from scratch or if they were a semi-finished product.

Participants also mentioned that they found it okay to eat candy on Saturdays if they did not snack on it all the time; in other words, eating unhealthily was considered fine if it was one day per week but doing it constantly might pose a problem. Some respondents acknowledged that they thought about what they eat but it was predominantly in positive terms such as eating before sports, how often one was supposed to eat if wanting to gain weight or build muscles, or thinking of one’s favourite dish, such as chicken breast and rice. No one spoke of counting calories or being afraid of eating too much. Negative feelings were rarely discussed during the focus groups. The boys seldom worried about what they ate, and if they did, it was usually worry about not eating enough: *“I’m tall and thin.”* (Fg D). Very few participants worried about eating too much of what was bad for you, with salt taking first place, followed by chocolate.

### “To be hungry or not. That is the question”

Most participants initially stated that they did not often feel hungry in general. Hunger was often related to the mornings or right before lunch, leaving them wondering whether they might have a sort of internal clock that makes them hungry. They also felt hungry after gym. However, several participants said that they almost never felt hungry, while some stated that they felt hungry constantly. One boy expressed that when they [presumably his caregivers] told him they would start dinner, “*then you start starving”* (Fg B). Even though they did feel hungry and did eat, many participants did not feel satisfied for very long: “*Even though I feel damn full because I’ve just eaten so much, I feel hungry five minutes later.*” (Fg D). Several boys did not feel sated after eating lunch in school and found that problematic. They discussed that all students get the same amount of food (e.g., five meatballs per student) instead of dividing it according to age, giving the 13-year-olds more to eat than the 7-year-olds. Moreover, there was sometimes simply not enough food in the cafeteria, which they found difficult. They suggested that potatoes should always be served as an extra dish to be able to reach satiation in school. One boy had a graded system for satiation:

*“It’s different… you can be full in different ways, you can be full so you don’t want to eat more if it’s not delicious, but if it’s delicious then… and you haven’t eaten for a long time, you can after all, eat until you are double-full.*

*Interviewer: how does double-full feel?*

*The tummy is completely full*

*Interviewer: and how long does that last?*

*About an hour…… then it passes.”* (Fg F)

### Boys DO care about appearances

There were varying opinions among the boys regarding the importance of physical appearance in relation to food, ranging from physical appearance being important to not caring at all. For some, it was abundantly clear that “good looks” was interpreted as a fit body: *“Yes, for me it is the case that the body is important but not the face and things like that, how it looks.”* (Fg A). Others were aware of the role that food played in their physical appearance:

*“So, food is sort of like this… many people think that ‘now I’m going to go and exercise, and I’ll look good’, but… the most important thing is the food.”* (Fg B).

One rather unexpected aspect of the discussion about food and physical appearance was views regarding the movement for body positivity. Participants thought that it might be risky to be obese and even though they felt it was helpful to be content with one’s looks and body, it could also be fatal to be severely overweight despite having a body-positive attitude:

*“And being satisfied with one’s appearance, if you are [obese], it can be incredibly dangerous, because you are on the verge of eating yourself to death.”* (Fg B).

### Dieting and weight gain

All participants knew of dieting, although some were unfamiliar with the word and seldom did they think of it. Only a small number of boys had considerations about weight loss, with one showing a strong interest in nutrition. The only time dieting was mentioned in school was when they spoke about the importance of eating in gym class or during other sport-related events. A general perception was that it was not common for boys to diet. Some theorized that if dieting was important to you, you should see the school psychologist. Opinions differed as to whether dieting was defined as just losing weight or if it meant losing weight by drastically cutting calorie intake. Dieting was related to general health and was considered difficult to implement: *“It’s hard to stop eating actually, it’s hard to stop.”* (Fg A).

Some participants suggested that dieting was related to age, with dieting being uncommon at their age but “…*Older ages surely do [it]”* (Fg F). Several boys also talked about the opposite of dieting or gaining weight. Some described being too thin and needing to eat more to build up strength.

*“Because I… I mean I eat so much, but I never gain weight.”* (Fg A)

*“… I worry like I’m eating too little.”* (Fg D)

A few of the boys who considered themselves ‘wispy’ or who had been wispy at a younger age stated that it was their fathers who had encouraged them to eat more:

*“…Dad wanted me to not be skinny like this…”* (Fg D).

### Disordered eating is a tricky matter

Not all boys knew what an ED was: “*You mean, you throw up and such?*” (Fg A). Moreover, neither EDs nor disordered eating were discussed or much thought about among them. Different ideas regarding the meaning of such illnesses were suggested, such as dieting, not being hungry, or vomiting everything that you eat. Participants in the younger groups also asked whether having an ED meant eating all the time or not wanting to eat at all. Not everything was framed negatively: “*the brain feels good when not eating*” (Fg E) and that “*the body…, or…, you sort of praise yourself when not eating*” (Fg E). Participants who were aware of EDs commented that they are probably more common than one would think and even though they did not know anyone in person, they speculated that there was probably always someone at every school. Despite this, there was some hesitation about whether it was a good idea to speak more of it in school, as it was considered a sensitive subject and might be a trigger for some people.

Participants described that what they had viewed on social media about eating and weight was all very negative. One group engaged in a lively discussion whether ED is considered a mental or a physical illness. One of the boys indicated that he had suffered from an ED. Otherwise, the general view was that it was something seen only on film and that only girls suffered from, or at least for the most part. Furthermore, participants reflected that girls might care more about their looks and be more open about suffering from eating problems.

*“I think that girls are more open about it, and you are more attentive to girls, like, if you notice that a girl, like, [very quiet], is down.”* (Fg D)

Some participants expressed scepticism about the subject, suggesting that some girls might use it for attention. They further suggested that it would be easier for a boy to hide an ED, although they never made clear in what way: *“… But in a guy, it feels like, you never see it…”* (Fg D).

## Discussion

This study aimed to shed light on adolescent boys’ perceptions of eating, weight, and food intake to contribute to further understanding of their understudied perspective. To systematize this discussion, Bandura’s social cognitive learning theory [38, 39] is used, a psychological framework that emphasizes the role of cognitive processes, observational learning, and the interplay between personal factors and the environment in shaping human behavior.

While all of the participating boys reported that they ate healthy food five days per week and ate unhealthy food about three days per week, one third of the sample was, according to the ISO-BMI scale [42], overweight or obese. In accordance with the second theme, “*Don’t worry, food makes you happy,”* the boys seemed rather unfazed by their (potential) weight issues. However, males tend to be overweight before showing signs of an ED [43, 44]. Social learning theory [38, 39] places a strong emphasis on self-efficacy, or a person’s confidence in their ability to “perform” a specific behavior. In this case, participants’ lack of worry about weight might be influenced by a high level of self-efficacy regarding their perceived ability to obtain and enjoy food in addition to knowing what food is good for them. Conversely, the participants also discussed the “body positivity” movement as being unhealthy, indicating that they do have a perception of how food can relate to one’s appearance in a negative way. According to Bandura [38, 39], a person’s self-perception and beliefs about their attractiveness can impact appearance-related behaviors. For example, someone with a positive body image might feel more comfortable and confident in their appearance choices, whereas someone with a negative body image might engage in behaviors aimed at changing their appearance or may experience lower self-esteem. There may be reason to question the ISO-BMI cut-off values, but it might also reflect that boys’– or at least those who participated in this study– do not care about being overweight. This might also be one of the reasons that the public health issues surrounding eating too little or too much has not been thoroughly explored among adolescent boys. If someone who has a problem does not recognize it, then is there really a problem? Bandura [38, 39] argued that people learn through observation of others; it is possible that these boys observed individuals who have a positive attitude towards food, eating, and their own weight (whatever it may be), which in turn influenced their own attitudes and behaviors. One possible source for this learning would be from family members. Previous research has indicated that eating at least one meal per day with family, which most participants in this study did, is a protective factor against ED diagnosis [17, 43].

Although participants were not specifically asked about their ethnicity, some indicated challenges with certain food-related terms during the interviews, implying they might not have been in Sweden for an extended period. Their answers to questions about happiness and food, such as that related to school lunch, indicated that some boys might have come from an environment where food is scarce. Their lack of worry about food might therefore have been influenced by positive cognitive appraisals of the newfound high food availability. However, they also mentioned that sometimes the school lunch did not fully sate them. The issue of sufficient nutritious food in schools was raised more than twenty years ago [45], but still seems to be an issue. Another aspect of concern among the boys was whether it was equitable for younger children to receive the same meatball or overall food ratio as the older children at the school canteen. Although it’s true that a larger body requires more energy, it’s important to acknowledge that younger children experience growth spurts demanding a considerable amount of energy. Could the underlying issue be that the established general ratios are inadequate for fulfilling the nutritional requirements of children overall?

*The intertwined relationship between food and one’s health* was discussed extensively among the boys. Two of the focus groups came from a school who had physical education on their schedule five times per week. Most of the other boys also spoke of playing different forms of sports or going to the gym and had good health literacy. They often discussed what food was good or bad for them, not only in terms of the *feelings* the food provoked but also in terms of its macro nutritional value, such as white bread being worthless for satiation or other foods not being rich in carbohydrates [46]. These results confirm past research indicating that adolescents are generally knowledgeable about healthy eating [47] and further emphasizes the importance of offering healthy (and sufficient) food during the school day. School lunches, which are served at all Swedish schools, tend to be more favorable than packed lunches, which makes it important that these lunches are nutritious [48, 49].

Participants focused primarily on physical health and feeling good until they were specifically asked about mental health. Mental health did not appear to be an oft-discussed or thought about topic amongst participants, in spite of the fact that adolescent mental well-being appear to decline among adolescents [50]. Education on mental health and well-being might therefore be another important area for schools to explore, as it is closely related to healthy eating and other lifestyle behaviors.

In discussions around the theme *“To be hungry or not. That is the question,”* some boys stated that they were hungry all the time but did not appear to express guilt or negative feelings about this. This contrasts with the *Boys DO care about appearances* theme, although this may be because they cared predominately about full body image. This might also have to do with role models [38, 39] on social media, a topic that goes beyond the scope of this study. Individuals with high self-efficacy regarding their appearances probably have a sense of confidence and belief in their ability to present themselves in a way that aligns with their desired image and feel capable of managing their appearance to meet their personal standards. However, it is important to note that Bandura’s theory does not address societal pressures, cultural influences, or the media’s role in shaping appearance ideals and standards.

As Nagata et al. [26] mentioned, the unique characteristics for boys regarding *dieting and weight gain* may be driven by the ideal body of muscularity and leanness. However, some boys did talk about anorexia nervosa, where the most common knowledge of the disease was the aspect of self-starvation. They reported that the brain “*feels good”* when not eating, which treads the ground of the perceptions of those in the early stages of anorexia. These statements are in line with earlier research showing that restrained eating, even at the clinical level, does not have a significant impact on adolescents’ psychological health, but can conversely improve it [14], at least in the short term. Disordered eating is, indeed, a tricky matter.

### Limitations

This study is not without its limitations. Although one of the researchers is a licensed teacher for the participants’ age group, it can be challenging to speak to a group of adolescents without prior familiarization. Certain groups and individuals were notably talkative, while others were less so. There was a potential risk that boys whose mother tongue was not Swedish might have been less engaged in the conversation, although no clear indications of this were evident. To encourage participation in focus groups and to create an environment for everyone to speak freely is demanding [33]. The focus group dynamics may also be particularly sensitive for adolescents who do not want to ‘out’ themselves in front of their peers. However, several studies have used focus groups to answer questions about similarly serious and demanding themes such as abortion or dating violence [51, 52]. Furthermore, the authors are experienced in conducting focus groups and tried to balance the discussion by making sure that everyone had the possibility to participate.

To enhance the trustworthiness of the study, we implemented several methodological measures and reflected on them, including: (1) Utilizing well-established research methods familiar to both researchers. (2) Acquiring a thorough understanding of the culture within the participating organizations. (3) Employing a convenience sample to mitigate potential researcher bias. (4) Ensuring participant honesty by allowing genuine willingness to participate and offering multiple opportunities to decline involvement, and (5) staying close to the participants’ perspectives by focusing on the manifest content of the data, among other strategies[53]. It’s essential to note that exploratory studies aim to deepen understanding within a specific target group rather than seeking generalization of the results [54].

The study’s findings may possess limited generalizability to diverse contexts; however, they can offer valuable insights into how adolescent boys perceive food and food consumption in various settings. Considering the strong influence of culture on food preferences and habits, it is important to consider social and cultural norms when interpreting the results. Relatedly, we did not inquire about the participants’ cultural identity, which could have potentially enriched our findings and offered a more nuanced understanding of their experiences regarding food and food intake.

Finally, one individual interview was collected which represents a different type of data collection compared to the planned focus group design and thus cannot be readily comparable to the information received from the focus groups. Yet, this interview did provide an important insight on the research question posed and did not deviate in this matter.

## Conclusions

While earlier research indicates that disordered eating among adolescent boys is a potentially serious public health issue, physically as well as mentally, our study indicated that the boys primarily focused on the physical aspects of eating and did not connect it to mental health issues unless asked explicitly. The notion that they did not find the school lunches satisfying is worthy of note, especially in Sweden, a country that prides itself on its school lunches. In all, the discussions about food and weight had a rather positive emphasis, again bringing the pathological perspective into questioning when it comes to measuring disordered eating among adolescent boys.

## Data Availability

The datasets generated and/or analysed during the current study are not publicly available because the participants were promised that the two researchers would keep all data to themselves. De-identified data is available from the corresponding author on reasonable request.
